# Voltage-Gated Calcium Channels and the Parity-Dependent Differential Uterine Response to Oxytocin in Rats

**DOI:** 10.1007/s43032-024-01765-8

**Published:** 2025-01-13

**Authors:** Korie Sondgeroth, Elisabeth Boyman, Riya Pathare, Maura Porta

**Affiliations:** 1 Department of Physiology, College of Graduate Studies, Midwestern University, Downers Grove, IL 60515 USA; 2 Chicago College of Osteopathic Medicine, Midwestern University, Downers Grove, IL 60515 USA

**Keywords:** Pregnancy, Oxytocin, Uterus, Contractility, T-type calcium channels, L-type calcium channels

## Abstract

The experience of pregnancy affects uterine function well beyond delivery. We previously demonstrated that the response to oxytocin is more robust in the uteri of proven breeder rats. This study investigates the contribution of T-type calcium channels (TTCCs) and L-type calcium channels (LTCCs) to the distinct response of virgin (V) and proven breeder (PB) rat uteri to oxytocin. Dose-inhibition responses to mibefradil (TTCC inhibitor) and verapamil (LTCC inhibitor) were conducted on isolated V and PB uterine strips. These experiments were followed by dose–response curves to oxytocin (10–10 to 10–5 M) in the presence of 10 µM of each inhibitor. Area-under-the-curve (AUC), amplitude, frequency, and duration of contractions were measured. V uteri generally showed a greater dependence on VGCCs, especially TTCCs. However, PB uteri exhibited a stronger frequency response to oxytocin. Blocking TTCCs had a more pronounced impact on the differential oxytocin response, particularly affecting the frequency component of contractions. The stronger frequency response in PB uteri may be due to a higher concentration of TTCCs in their myometrial pacemaker cells. This study provides supporting evidence that pregnancy induces lasting changes in uterine calcium handling. Our findings suggest that TTCCs play a more important role than LTCC in the parity-dependent differential response to oxytocin. The impact of ORAI and TRP channels still needs to be evaluated, to gain a more comprehensive understanding of the relative impact of voltage-gated calcium channels vs. storage-operated calcium entry channels on this phenomenon.

## Introduction

The experience of pregnancy, with its hormonal and mechanical challenges, drastically affects the overall female physiology. These challenges are likely to leave an imprint, in the form of epigenetic modifications, on the function of various organs, especially the uterus and the reproductive system. In our previously published study [1], we demonstrated that, after pregnancy, some contractile properties of uterine tissues are permanently changed. Elucidating the mechanisms underlying these differences will increase our understanding of various epidemiological findings regarding risk factors associated with parity, such as uterine atony and preterm birth. Our earlier experiments with rat uteri demonstrated that oxytocin caused a more pronounced contractile response in tissues from females with a history of pregnancy (proven breeders, PB) compared to virgin (V) animals. Interestingly, this disparity between PB and V samples was virtually eliminated when the extracellular calcium (Ca^2+^) concentration was lowered from 2.5 mM to 10 μM. This suggested that the differential response to oxytocin between V and PB depended on calcium entry. Thus, we speculated that the expression and/or function of either voltage-gated Ca^2+^ channels (VGCC) or store-operated calcium entry (SOCE) elements might be altered by the pregnancy experience and mediate the differences observed in the two groups. In the present study, we explored the contribution of VGCC to this phenomenon.

It’s worth noting that VGCCs are not directly altered by oxytocin. However, since oxytocin can modify membrane potential via PCK-stimulated phosphorylation of the Slo 2.1 sodium-dependent potassium leak channel, VGCC function is indirectly affected as well [2].

T-type Ca^2+^ channels (TTCC) are low-voltage activated channels located on the plasma membrane of excitable and non-excitable cells [3]. They participate in the development and regulation of various tissues, including neurons, cardiac and smooth muscles, and endocrine glands [4, 5] [6–8]. TTCCs require only a moderate level of depolarization to activate (above −70 mV) and produce short-lived (***transient***) and small (***tiny***) currents [5]. They quickly inactivate at voltages above −40 mV and require hyperpolarizing conditions to fully recover. These properties are consistent with their role as depolarization triggers in certain tissues (e.g.: pacemaker cells). In other tissues, the small inward currents generated by TTCCs are thought to be key activators of Ca^2+^-dependent ion channels, enzymatic processes, and gene expression. Different tissues express different isoforms of TTCCs. Three main isoforms have been identified so far (Ca_v_3.1, Ca_v_3.2, and Ca_v_3.3; [9]).

Electrophysiological and RT-PCR studies have demonstrated the presence of TTCCs in uterine tissue from humans and rats [10–12]. Specifically, both the Ca_v_3.1 and Ca_v_3.2 subunits have been found in pregnant rat myometrium [13]. The function of TTCCs in the uterus seems to be the activation of spontaneous phasic contractions [14]. These spontaneous contractions require a process of slow depolarization like that of pacemaker cells [14]. Thus, it is not surprising that TTCCs would contribute to this slow depolarization in the myometrium just as in other smooth muscles and the heart.

TTCCs have been implicated in the etiology of different pathologies, including Parkinson’s disease [15] and cancer [16]. Activation of TTCC by lipopolysaccharides from an infectious agent has been connected to increased myometrial contractility during pregnancy and preterm birth [17].

The limited availability of selective blockers has hindered pharmacological studies of TTCCs. Although alternative options have been tried over the years, Nichel and mibefradil are still commonly used for inhibition studies. We chose to use mibefradil, a benzimidazole-substituted tetralin derivate, for its high affinity for the channel, although its selectivity is less than ideal [14, 18]. Mibefradil binds the central portion of the pore domain. In so doing, it causes the displacement of a phospholipid component normally associated with the channel pore, just below the selectivity filter. This displacement reduces Ca^2+^ influx [19]. On the other hand, mibefradil can also inhibit L-type Ca^2+^ channels and some K^+^ channels. This will bear consideration [20]for our data interpretation.

L-type Ca^2+^ channels (LTCC) are voltage-gated membrane channels that activate at higher potentials than TTCC (−10 mV) and elicit large, long-lasting inward currents. These currents produce significant Ca^2+^ entry, which in turn can produce sustained action potentials in ventricular myocytes as well as activate contraction in cardiac and smooth muscles. LTCCs inactivate via a slow, voltage-dependent mechanism and by a fast, Ca^2+^ entry-dependent mechanism. Of the three subunits, α,β and γ, that constitute the channel, α forms the pore, while β is responsible for the voltage-dependent inactivation (δ controls trafficking and channel regulation, [20, 21]. The Ca^2+^ entry-dependent inactivation is mediated by a molecule of calmodulin closely associated with the channel [20, 21]. In neurons and endocrine cells, LTCCs are involved in exocytotic processes[22, 23]. Like TTCCs, LTCCs also regulate gene expression. In skeletal muscles, LTCCs’ main role is to activate ryanodine receptor channels and induce Ca^2+^ release from the sarcoplasmic reticulum (voltage-dependent Ca^2+^ release) [24]. In smooth muscles, LTCC carry a large component of the Ca^2+^ for contraction[2, 25, 26]. The intensity of these LTCC Ca^2+^ currents depends on the number of channels as well as on the degree of regulation of various hormones, metabolic conditions, and cyclic nucleotides [2].

Many pharmacological inhibitors of LTCC have been discovered and utilized over the years: dihydropyridines (e.g.: nifedipine), benzodiazepines (e.g.: diltiazem), and phenylalkylamines (verapamil). An additional class of inhibitors is derived from naturally occurring peptides (e.g.: ω-agatoxin IIIA from the funnel spider’s venom). We selected verapamil rather than dihydropyridines for our experiments, because of its better resistance to photodegradation. Verapamil is a common drug used to treat arrhythmias [27, 28]. It has established therapeutic uses for tachycardic atrial fibrillation[29] and some forms of ventricular tachycardia [30]. Verapamil induces inhibition of LTCC by crossing the plasma membrane and interacting with a region of the pore close to the intracellular side [31]. Like the majority of the L-type channel inhibitors, verapamil lacks specificity. It can also interact with TTCC as well as with some potassium channels [32–35].

Applying mibefradil and verapamil to dissected strips from V and PB uteri and studying their response to oxytocin, we demonstrated that parity affects the availability of VGCC. In addition, we found that TTCC currents contribute to the stronger frequency response to oxytocin in PB uteri.

## Materials and Methods

### Animal Procurement and Care

This study adhered to protocols approved by the Midwestern University (MWU) Institutional Animal Care and Use Committee (IACUC; protocol #2758) and followed National Institutes of Health (NIH) guidelines for laboratory animal welfare. Non-pregnant female CD Sprague Dawley rats, aged 16 weeks, were obtained from Charles River. Animals were housed in pairs with unrestricted access to food and water under standard laboratory conditions.

To minimize hormonal variability impacting myometrial contractility, tissue collection occurred during the proestrus phase of the estrous cycle. Daily vaginal cytology, performed at a consistent time (~ 9:30 AM) for 10–12 days, identified the proestrous stage based on criteria established by Marcondes et al. [36]. We selected an experimental age of 18 weeks ± 1 week. This represents the youngest age at which females from Charles River’s breeding program can deliver a second litter. This age standardization minimizes potential age-related variations in myometrial characteristics, including contractile protein expression, immune system components, lipid transport, and metabolism. Future studies will explore how parity (number of litters) influences uterine function during pregnancy. Consistent age across all groups facilitates more accurate comparisons of results.

Following euthanasia via CO_2_ inhalation and cervical dislocation, the uterine horns of proestrus rats were excised and immediately immersed in chilled, oxygenated, low-Ca^2+^-modified Krebs-Heinseleit solution (in mM: NaCl 117, KCl 4.7, NaHCO_3_ 25, MgCl_2_ 1.2, KH_2_PO_4_ 1.2, CaCl_2_ 0.01, glucose 11; pH 7.4) for storage on ice and subsequent dissection. It has been reported that prolonged hypoxia (4–24 h) can alter motility and protein expression of the non-pregnant uterus. While acknowledging the potential impact of hypoxia on the myometrium, we are confident that the effect of the CO_2_ inhalation on the contractility and protein expression is minimal given the relative brevity of the exposure and the prompt collection and handling of the tissues [37, 38]. Each horn was swiftly opened lengthwise along the mesometrial border. Endometrial debris were gently cleared from the internal surface of the horns using a cotton swab. One horn was weighed, immediately frozen using liquid nitrogen, and stored at −80 C for subsequent molecular investigations. The remaining horn was dissected into six longitudinal (and occasionally circular) strips of 3 mm × 10 mm dimensions for contractility assessments. We are aware that the hypoxic conditions generated by the exposure to CO_2_.

### Reagents

Oxytocin, mibefradil, and verapamil were sourced from Cayman Chemical Company (Ann Arbor, MI, USA), while all remaining reagents were purchased from Sigma Aldrich (St. Louis, MO, USA).

### Contractility Measurements

Individual uterine strips were mounted in 10 ml chambers (Myobath II, World Precision Instruments, Sarasota, FL) within a physiological Krebs-Heinseleit bath (in mM: NaCl 117, KCl 4.7, NaHCO_3_ 25, MgCl_2_ 1.2, KH_2_PO_4_ 1.2, CaCl_2_ 2.5, glucose 11; pH 7.4; also referred to as regular Krebs solution). Bath conditions were maintained at 37 °C with continuous oxygenation (95% O2, 5% CO2). Each sample was placed under 0.5 g of tension. Force transducers (WPR) connected to an IX228/S bridge amplifier (iWorx, Dover, NH) enabled force of contraction measurement. Data acquisition was managed using LabScribe software (iWorx). Under these conditions, spontaneous and regular contractions were observed. Strips failing to demonstrate sufficient contractile activity within 15 min were excluded.

Following a 30-min equilibration, in the dose–response to oxytocin experiments, a 40 mM KCl challenge was used to establish maximal activation. Contractility obtained under this condition served as a normalization reference for later measures. Three successive 10-min washes with regular Krebs solution effectively removed residual KCl and restored baseline contraction patterns. Dose–response experiments were then initiated. The KCl challenge was omitted in the inhibition experiments with voltage-gated calcium channel (VGCC) inhibitors.

LabChart 7 software (ADInstruments, Colorado Springs, CO) was employed to record and analyze uterine contractions. Contractility was reported as the Area Under the Curve (AUC) and determined over the initial 5-min interval following drug administration. Amplitude, frequency, and duration parameters were also assessed. Contraction duration was measured based on the time interval on the X-axis between the points corresponding to 50% of the maximum amplitude.

### Statistics

A sigmoid dose–response curve was generated via the standard least-square fit method to model the data.

$$y=\text{min}+\frac{max-min}{1+{10}^{(\text{log}EC50-x)}}$$

Differences in logEC50, maximal, minimal, and range responses to oxytocin were analyzed utilizing a two-tailed t-test. A *P*-value of < 0.05 was the threshold for statistical significance. SigmaPlot 13 was the software used for all statistical analyses.

## Results

### Inhibition of Spontaneous Activity by Mibefradil

**Fig. 1:**
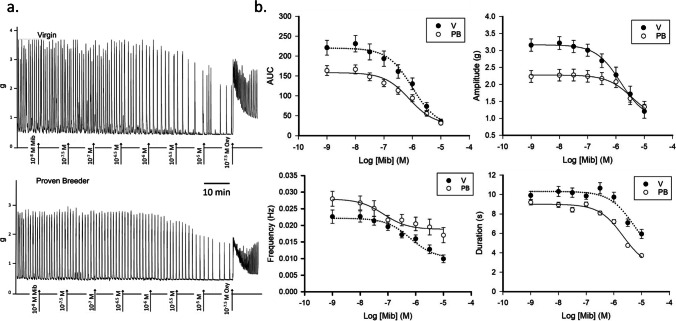
Dose-inhibition response to mibefradil. **a.** Sample traces recorded in uterine samples from virgin (top panel) and proven breeders (bottom panel) female rats. **b.** Dose inhibition response curves summarizing data obtained from experiments conducted on 4 virgin (V, black circles) and 4 proven breeder (PB, white circles) rats (6 uterine strips/rat). The top left panel shows the overall contractility decline under increasing doses of mibefradil, represented by the area under the curve measured over 5 min. For this experiment, no normalization was conducted. The top right panel shows amplitude changes. The bottom left panel represents mibefradil’s impact on contraction frequency. The bottom right panel shows changes in duration with increasing amounts of the T-type channel inhibitor

In the first step of our investigation, we evaluated the inhibition of spontaneous uterine contraction by increasing doses (10 nM to 10 μM) of the t-type calcium channel inhibitor mibefradil. The dosing range was selected based on the observed uterine strip contractile response. 10 nM was the smallest drug concentration at which an effect was revealed. Beyond 10 µM, no further activity reduction was detected. Virgin (V) and proven breeder (PB) myometrial strips were suspended with a passive tension of 0.5 g in Krebs solution containing 2.5 mM CaCl_2_ (regular Krebs). Four parameters were quantified: area under the curve (AUC), amplitude, frequency, and duration. Figure 1.A displays sample traces of the experiment, while Fig. 1.B shows the relative summary curves (for both V and PB, rat *n* = 4). The curve parameters are reported in Table 1.A. Typically, area-under-the-curve data are reported normalized for the AUC recorded for a challenge with 40 mM KCl. Treatment with KCl induces massive depolarization of the plasma membrane and thus maximal contraction of the strips. The resulting AUC reflects the activation of all the muscle fibers of a particular strip. Normalization by the AUC in 40 mM KCl allows us to account for unintended variations between strips. However, we previously observed [1] that spontaneous contractility is considerably reduced by the washes that add and remove the KCl. Thus, for both the mibefradil and verapamil inhibition experiments, we chose to forgo the KCl challenge and sacrifice our ability to account for uneven sample characteristics to get a clearer estimate of the full inhibitory effect of these drugs on the two groups. The raw AUC data show that the spontaneous activity of V samples was higher than that of PB rats (*P* < 0.001, see Table 1), which is consistent with our previous observations [1]. The mibefradil-induced inhibition range (Max–Min) was larger in V than in PB strips (*P* < 0.001, see Table 1). Some, albeit limited, contractile activity was maintained even at the highest mibefradil concentration (10 µM). At this highest dose of inhibitor, no difference in AUC was detected between sample types. Also, the IC50 was similar. The amplitude of spontaneous contractions (Table 1.B.) and the range of the mibefradil-induced inhibition were higher in V samples than in PB samples. In 10 µM mibefradil, no differences in amplitude were recorded between the groups. The IC50 calculated for the amplitude curve was also similar in V than in PB.

**Table 1:** Dose inhibition response to mibefradil and verapamil parameters

A. Inhibition AUC Curve Parameters (g*min)							
Parameter	Mibefradil			Verapamil			
	V	PB	P	V	PB	P	
IC50	−6.0 ± 0.1	−6.1 ± 0.1	0.6	−6.9 ± 0.1	−7.0 ± 0.1	0.5	
Max	220 ± 6*	158 ± 5	4E^−10^	145 ± 4*	107 ± 4	2E^−8^	
Min	22 ± 12	27 ± 11	0.7	3 ± 3	3 ± 3	1	
Range	198 ± 13*	131 ± 11	3E^−4^	142 ± 5*	100 ± 5	4E^−7^	
B. Inhibition Amplitude Curve Parameters (g)							
Parameter	Mibefradil			Verapamil			
	V	PB	P	V	PB	P	
IC50	−5.55 ± 0.06	−5.4 ± 0.1	0.1	−6.14 ± 0.07	−6.33 ± 0.08	0.8	
Max	3.17 ± 0.03*	2.28 ± 0.03	3E^−4^	3.01 ± 0.06*	2.08 ± 0.05	1E^−14^	
Min	0.96 ± 0.08	0.96 ± 0.09	0.9	0.07 ± 0.08	0.03 ± 0.08	0.7	
Range	2.21 ± 0.08*	1.32 ± 0.09	4E^−9^	2.94 ± 0.1*	2.07 ± 0.09	6E^−8^	
C. Inhibition Frequency Curve Parameters (Hz)							
Parameter	Mibefradil			Verapamil			
	V	PB	P	V	PB		P
IC50	−6.4 ± 0.2	−5.9 ± 0.06	0.1	−5.9 ± 0.1	−6.2 ± 0.2		0.2
Max	0.022 ± 0.001	0.028 ± 0.001*	0.02	0.0104 ± 0.0003	0.0155 ± 0.0008*		4E^−6^
Min	0.010 ± 0.001	0.017 ± 0.001*	0.01	0.001 ± 0.001	0.001 ± 0.001		0.9
Range	0.014 ± 0.001*	0.011 ± 0.001	0.05	0.0094 ± 0.003	0.014 ± 0.001*		0.002
D. Inhibition Duration Curve Parameters (s)							
Parameter	Mibefradil			Verapamil			
	V	PB	P	V	PB		P
IC50	−5.4 ± 0.2	−5.9 ± 0.1*	0.03	−6.79 ± 0.07	−7.21 ± 0.08*		1E^−3^
Max	10.0 ± 0.3	9.6 ± 0.2	0.2	10.7 ± 0.2	10.1 ± 0.3		0.2
Min	5.9 ± 0.4*	3.7 ± 0.1	3E^−5^	2.3 ± 0.2	2.3 ± 0.3		0.8
Range	4.1 ± 0.5	5.9 ± 0.2*	0.02	8.4 ± 0.3	7.8 ± 0.3		0.1

The frequency of spontaneous contractions was stronger in PB than in V (Table 1.C) and was consistently higher in PB strips during the entire sequence of mibefradil doses. Thus, the curve minimum was also significantly higher in PB samples. The range of inhibition was larger in V than in PB. This suggests that T-type channel inhibition might impair the formation of calcium-calmodulin complexes more in V strips in PB. The differences in IC50 did not reach significance.

In terms of durations, V and PB did not differ in the absence of inhibitors, but other curve parameters were significantly different (Table 1.D). In the presence of 10 µM mibefradil, the minimal duration recorded was higher in V than in PB (5.9 ± 0.4 vs 3 ± 0.1 s, *P* < 0.001). Thus, PB strips were more affected by the treatment than the V strips. 50% inhibition was achieved with a lower mibefradil concentration for PB than V.

At the end of the dose-inhibition experiment, the uterine strips were treated with 50 nM oxytocin. This caused a reactivation of contractility that appeared 33% stronger in V than in PB (403 ± 37 vs 294 ± 19 g*s respectively, *P* < 0.001). This was somewhat unexpected since typically PB strips are more responsive to oxytocin (Fig. 5.A top panel, Table 3). Amplitude/frequency/duration analysis revealed that the main determinant of the stronger response of V was the event duration, which was significantly longer in V than in PB (9.0 ± 0.4 s vs 6.1 ± 0.1 s, *P* < 0.001). Hence, 50 nM oxytocin restored the duration to the pre-mibefradil levels in V but not PB samples. Frequency, which appeared to be the main player in the stronger response to oxytocin in the absence of mibefradil [1] increased in equal measure in the two groups here.

### Inhibition of Spontaneous Activity by Verapamil

**Fig. 2:**
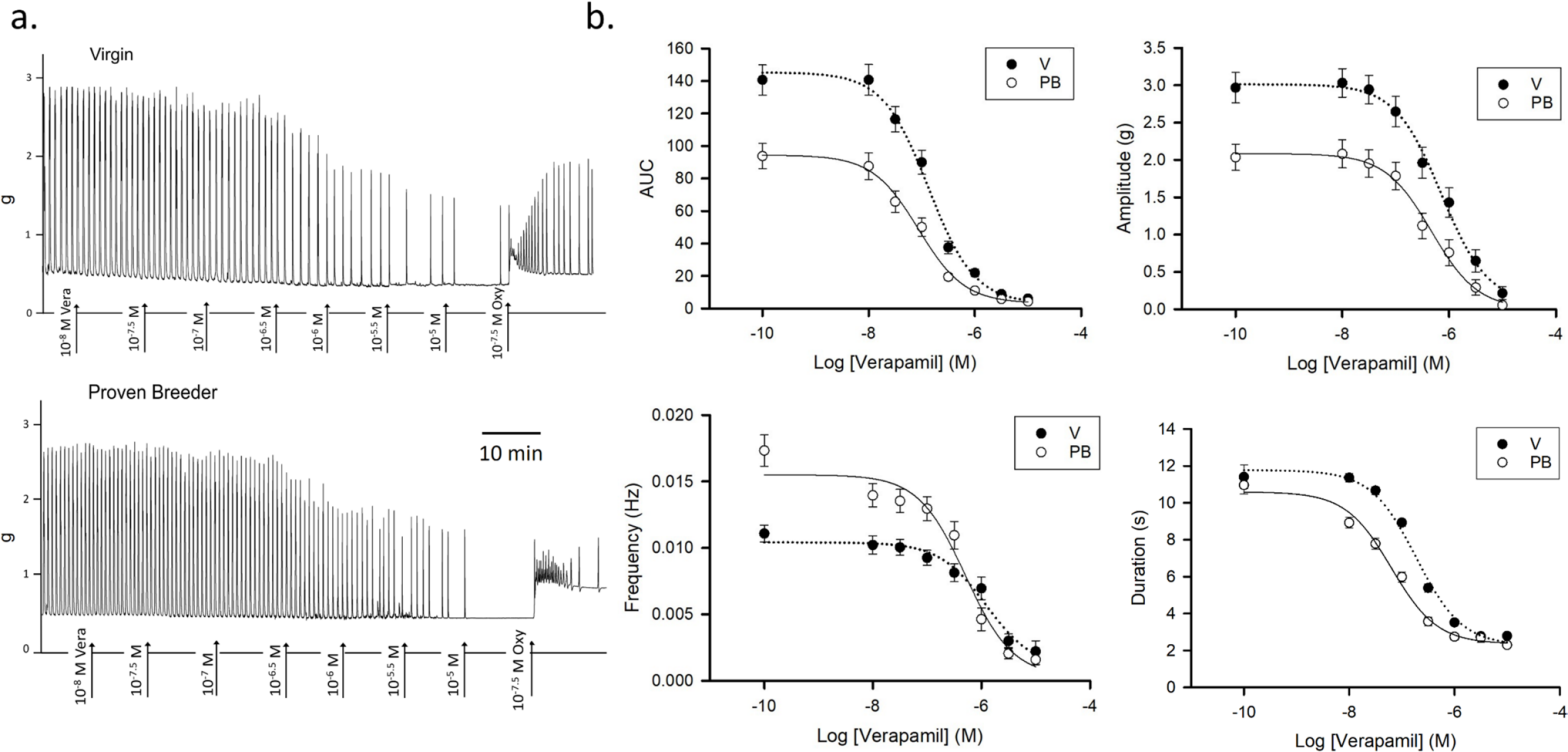
Dose-inhibition response to verapamil. **a.** Sample traces recorded in uterine samples from virgin (top panel) and proven breeders (bottom panel) female rats. **b.** Dose inhibition response curves summarizing data obtained from experiments conducted on 4 virgin (V, black circles) and 4 proven breeder (PB, white circles) rats (6 uterine strips/rat). The top left panel shows the overall contractility decline under increasing doses of verapamil, represented by the area under the curve measured over 5 min. For this experiment, no normalization was conducted. The top right panel shows amplitude changes. The bottom left panel represents verapamil’s impact on contraction frequency. The bottom right panel shows changes in duration with increasing amounts of the L-channel inhibitor

The inhibition of spontaneous V and PB uterine contractions by increasing doses (10 nM to 10 µM) of verapamil was then also evaluated. The dosing range once again was selected based on the observed strip response. As before, the strips were suspended with a passive tension of 0.5 g in Krebs solution containing 2.5 mM CaCl_2_ (regular Krebs), then treated with the L-type calcium channel inhibitor. As in the mibefradil experiments, to maximize the initial spontaneous activity of the uterine samples we did not conduct the challenge with 40 mM KCl. Figure 2.A shows sample traces of the experiment and Fig. 2.B the summary curves for AUC, amplitude, frequency, and duration (for both V and PB, rat *n* = 4). The curves’ parameters are listed in Table 1. Raw AUC showed once again a stronger spontaneous activity in V. Maximal concentrations of verapamil (10 µM) almost completely inhibited activity in both groups. The Max–Min Range was larger in V strips. IC50 was similar in V and PB (Table 1.A). The Amplitude curves follow the trend of the raw AUC curves, with significantly larger values for spontaneous values (= Max) and range in V samples (Table 1.B). The spontaneous frequencies once again were higher in PB. The inhibition achieved by 10 µM reduced frequency to levels close to zero in both groups. The inhibition range therefore appeared larger in PB than in P (Table 1.C.). No significant difference in IC50 was detected. The duration curves under verapamil were somewhat similar in the two groups. Spontaneous duration was similar in the two groups. Since high levels of verapamil abolished most contractions, the minimum duration was very low and essentially identical in both V and PB samples. Hence, the inhibition ranges did not differ significantly. However, the calculated IC50s resulted to be moderately different (Table 1.D), with PB strips event durations being inhibited more effectively than V strips (−7.21 ± 0.08 in PB vs −6.79 ± 0.07, *P* = 0.001).

Thus, inhibition of spontaneous contractile activity by mibefradil and verapamil featured similar characteristics, with amplitude accounting for most of the AUC differences noted between V and PB in both cases.

As in the inhibition by mibefradil experiment, after the last dosage of verapamil, we added 50 nM oxytocin, which reactivated contractility. In the verapamil case, the oxytocin-induced activation was not as intense as in the mibefradil case. Treatment with oxytocin after 10 µM verapamil restored AUC only to 50% of V spontaneous levels (109 ± 8 vs the initial 221 ± 18 g*s) and 80% of PB spontaneous levels (134 ± 9 vs the initial 163 ± 12 g*s). Instead, treatment with oxytocin after 10 µM mibefradil caused AUC to double the spontaneous contractility levels in both V and PB samples (Fig. 5.A top panel and Table 3). This is not surprising, considering that, with 10 µM verapamil, the inhibition of contractility had been complete. Another difference from the mibefradil inhibition experiment is that PB responded twice as robustly as V strips to this single high dose of oxytocin (*P* < 0.001). This result is consistent with previous experiments with oxytocin and no VGCC inhibitors.

Amplitude was increased by oxytocin to similar values (*P* = 0.5) in V and PB, thus it does not seem to play a role in this divergence. Duration in V strips grew more than in PB strips (7.2 s vs. 5.4 s, *P* < 0.001) after the oxytocin treatment. This aligned with the duration data obtained in the 10 µM mibefradil + 50 nM oxytocin study. However, event frequency was twice as high in PB than in V (*P* < 0.001) samples. Thus, frequency variations seem to drive the predominant response to oxytocin in PB. These findings match the data obtained in our earlier experiments with oxytocin alone [1]. The fact that frequency did not seem to play a lead role in the differential response to oxytocin in the mibefradil experiments was puzzling. Thus, to explore this aspect more thoroughly, we run dose responses to oxytocin in the presence of a fixed amount of VGCC inhibitor.

### Response to Oxytocin in 1 $\mu$M Mibefradil

**Fig. 3:**
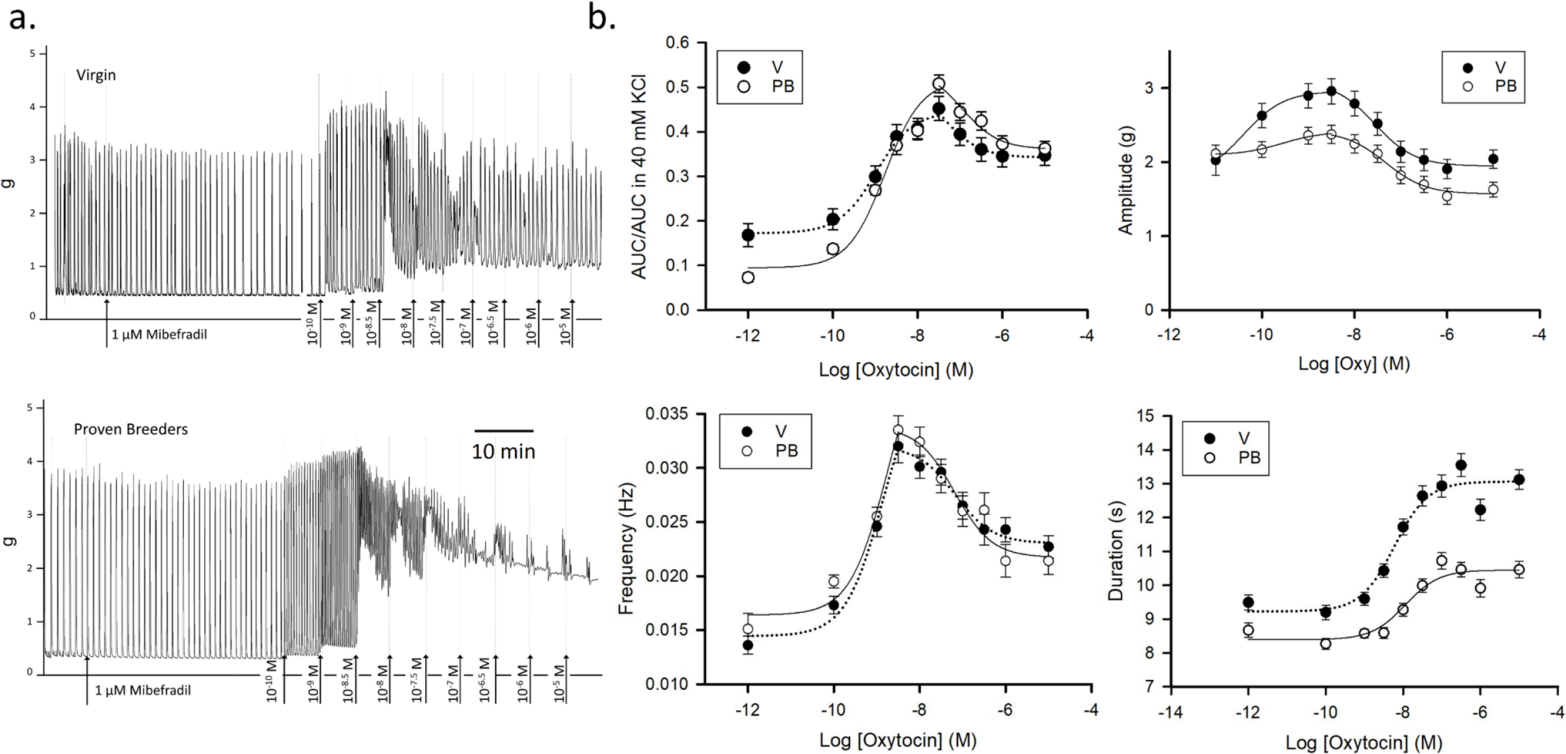
Dose–response to oxytocin in 1 μM mibefradil. **a.** Sample traces recorded in uterine samples from virgin (top panel) and proven breeders (bottom panel) female rats. **b.** Dose–response curves summarizing data obtained from experiments on 5 virgin (V, black circles) and 5 proven breeder (PB, white circles) rats (4–6 uterine strips/rat). Top left panel: overall contractility dose response to oxytocin in the presence of mibefradil (area under the curve over 5 min normalized by the area under the curve over 5 min of the 40 mM KCl challenge. Top right panel: contraction amplitude. Bottom left panel: contraction frequency. Bottom right panel: contraction duration. In the presence of 1 μM mibefradil, spontaneous activity and response to oxytocin were reduced. Dose–response curves for AUC, amplitude, and frequency displayed a hormetic trend. The AUC minimum was smaller and the maximum higher in PB than in V. This is reflected in the frequency curves and resembles the findings in the absence of mibefradil [1]. The amplitude and duration were steadily higher in V than in PB samples throughout the experiment

To test the hypothesis that T-type channels contribute to the differential response of V and PB uteri to oxytocin stimulation we preconditioned V and PB uterine strips with 1 μM mibefradil for 30 min. We were also interested in clarifying whether the effects of oxytocin on the frequency of PB strips are indeed suppressed by mibefradil as suggested by the previous experiment. In these experiments, all AUC values were normalized to the values obtained from a 40 mM KCl challenge. Figure 3.A displays sample traces, while Fig. 3.B the summary curves for AUC/AUC in 40 mM KCl, amplitude, frequency, and duration (for both V and PB, rat *n* = 4).

Except for duration, all the curves obtained in this experiment display a hormetic, bell-shaped trend. For example, the AUC/AUC in 40 mM KCl curve peaked at 50 nM oxytocin, then descended. In the presence of 1 μM mibefradil and no oxytocin, PB strips contracted less robustly than V strips (AUC/AUC in 40 mM KCl 0.09 ± 0.03 vs. 0.17 ± 0.01 in V, *P* = 0.02). At the peak of the response to oxytocin (Max), contractility was slightly stronger in PB than in V strips, and so was the range (Max–Min). The range of the descendent branch of the dose–response curve was similar in the two groups. No difference was found in the V and PB EC50 for oxytocin in both the ascending and descending branches.

The amplitude curve peaked at 5 nM oxytocin. V samples maintained a greater amplitude than PB at all oxytocin concentrations. The range of the oxytocin response was also more marked in V in the ascending and descending branches of the curve. V strips amplitude increased by 42% vs 15% in PB strips (0.87 ± 0.03 vs 0.31 ± 0.04 g, *P* < 0.0001).

The hormetic trend of amplitudes was previously observed in dose–response to oxytocin in the absence of VGCC inhibitor preconditioning. Yet, in those experiments, the minima were twice as high as in this case and the curve peaked at 50 nM oxytocin. The EC50 of the ascending branch of amplitude was significantly smaller than the EC50 of the AUC ascending branch (*P* < 0.001), while the EC50 of the descending branch was consistent between AUC and amplitude.

Frequency also peaked at 5 nM oxytocin. However, no significant difference was detected between the two groups. This result was consistent with the dose-inhibition response to mibefradil + 50 nM oxytocin experiments, in which we noted similar frequency in the two samples. The EC50 of the frequency curves matched the EC50 of the AUC curves. The lack of difference in frequency is the most remarkable finding of this experiment, as frequency was previously identified as the driving factor for the differential response to oxytocin associated with parity [1].

The duration curves show a stronger response to oxytocin in V than in PB after pretreatment with 1 µM mibefradil. Event duration before the addition of oxytocin was similar in 1 µM mibefradil and untreated samples. Duration of V contractions increased by 42%, in PB by 25% (3.9 ± 0.4 vs 2.0 ± 0.2 s respectively, *P* < 0.0001). The maximal response elicited by oxytocin exceeded both the spontaneous duration and the duration in 10 µM mibefradil + 50 nM oxytocin.

### Response to Oxytocin in 1 $\mu$M Verapamil

**Fig. 4:**
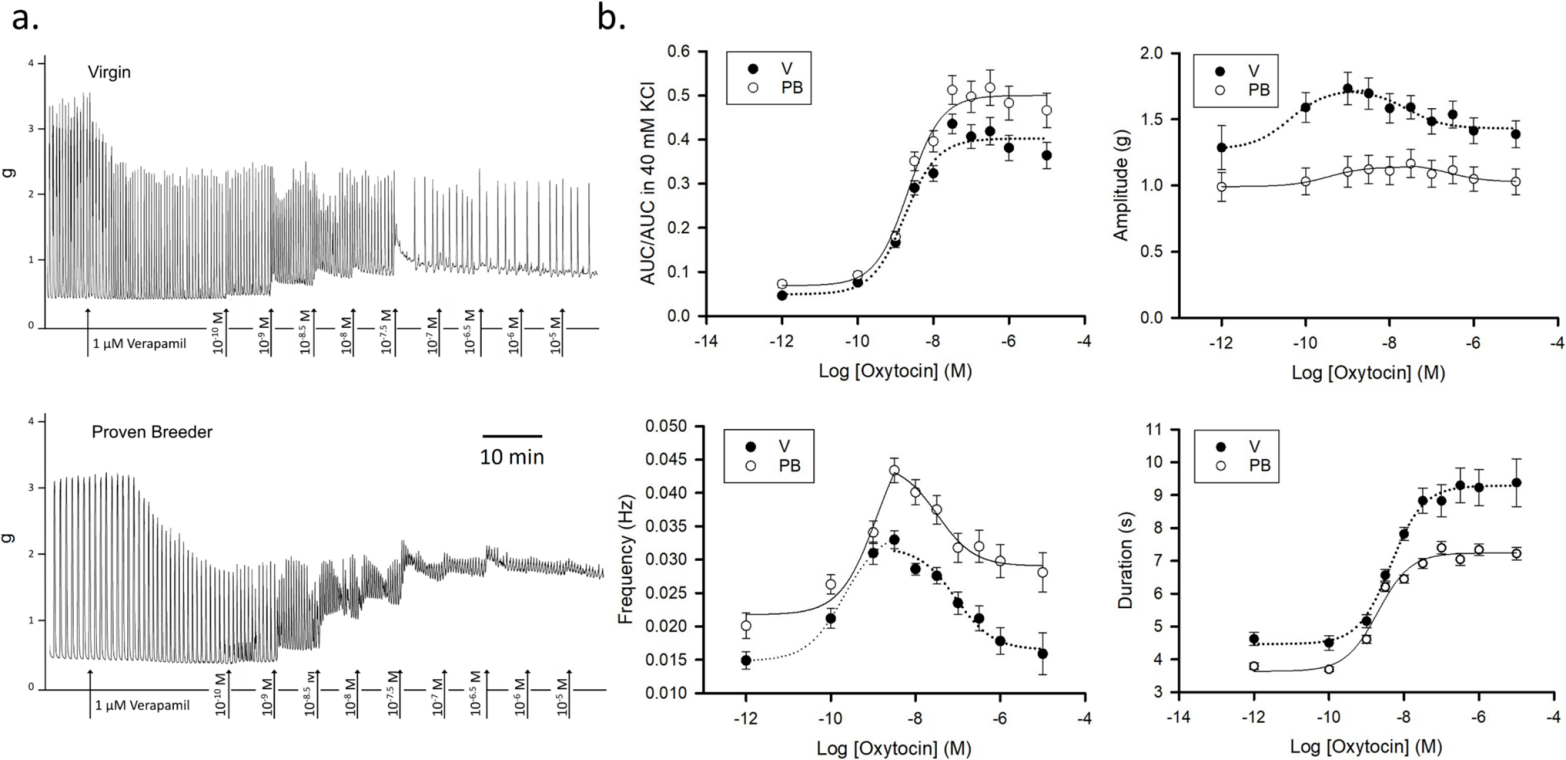
Dose–response to oxytocin in 1 μM Verapamil. **a.** Sample traces recorded in uterine samples from virgin (top panel) and proven breeders (bottom panel) female rats. **b.** Dose–response curves summarizing data obtained from experiments on 4 virgin (V, black circles) and 4 proven breeder (PB, white circles) rats (4–6 uterine strips/rat). Top left panel: overall contractility dose response to oxytocin in the presence of verapamil (area under the curve normalized by the area under the curve of the 40 mM KCl challenge. Top right panel: contraction amplitude. Bottom left panel: contraction frequency. Bottom right panel: contraction duration. In the presence of 1 μM verapamil, spontaneous activity and response to oxytocin were reduced. Amplitude and frequency but not AUC displayed a hormetic trend. The AUC minimum was smaller and the maximum higher in PB than in V. This is reflected in the frequency curves as with mibefradil and in the absence of VGCC blockers [1]. The amplitude and duration were steadily higher in V than in PB samples throughout the experiment

To evaluate the impact of L-type channel inhibition on the response to oxytocin, we preconditioned V and PB uterine strips with 1 μM verapamil for 30 min. Figure 4.A displays sample traces, Fig. 4.B the summary curves for AUC/AUC in 40 mM KCl, amplitude, frequency, and duration (for both V and PB, rat *n* = 4). The AUC/AUC in 40 mM KCl curves show a stronger contractile response from PB strips, with a trend closely resembling the dose–response to oxytocin in the absence of VGCC inhibitors from earlier experiments. A single sigmoidal model adequately captured the minor hormesis observed in the curve. This contrasts with the more pronounced hormetic effect of mibefradil preconditioning, which necessitates a more complex model for fitting.

The maximal response to oxytocin for both groups was roughly half the one recorded without VGCC inhibitors [1] and comparable in intensity to the one recorded in 1 μM mibefradil. The initial treatment with verapamil greatly inhibited phasic contractions, so that no difference was detected in the two groups (*P* = 0.5). However, both maximal response and range were higher in PB. The calculated oxytocin EC50s did not differ.

The amplitude curve had a bell-shaped trend. In the ascending branch, the minimum was similar in the two groups, due to the strong inhibition of calcium entry by verapamil. However, the maximal amplitude and the range of amplitude increase elicited by oxytocin were higher in V strips (Table 2.B). V and PB strips gained 35% and 15% of their initial amplitude respectively (from 1.27 ± 0.04 to 2.72 ± 0.03 g in V, from 0.99 ± 0.02 to 1.14 ± 0.01 g). In the previously published oxytocin experiment with no VGCC inhibitors [1], the range of amplitude increase was higher in PB (+ 59%, from 2.2 ± 0.01 to 3.5 ± 0.1 g) than in V (+ 26%,from 3.12 ± 0.04 to 3.94 ± 0.03 in V,). In addition, with verapamil, the V amplitude reached its peak at 5 nM oxytocin, while the PB amplitude at 50 nM. This was reflected in the significantly different EC50 of the ascending branch. In the descending branches, V amplitude decreased by 20% from its maximum, PB amplitude by 13% (0.29 ± 0.05 g vs 0.13 ± 0.06, *P* = 0.02). The EC50s of the descending branches were comparable and similar to the EC50s of the amplitude curves in mibefradil pretreated samples.

**Table 2:** Dose response to oxytocin parameter

A. Curves parameters for dose response to oxytocin in 1 μM Mibefradil												
			Ascending Branch									
Parameter	AUC/AUC in 40 mM KCl			Amplitude (g)			Frequency (Hz)					Duration(s)
	V	PB	P	V	PB	P	V	PB	P	V	PB	P
EC50	−8.9 ± 0.1	−8.7 ± 0.2	0.4	−10.3 ± 0.1	−9.7 ± 0.2	0.06	−8.4 ± 0.4	−8.5 ± 0.3	0.8	−8.2 ± 0.2	−7.9 ± 0.2	0.3
Min	0.17 ± 0.01*	0.09 ± 0.03	0.02	2.03 ± 0.02	2.10 ± 0.02	0.3	0.014 ± 0.001	0.016 ± 0.002	0.4	9.2 ± 0.3	8.4 ± 0.2	0.2
Max	0.46 ± 0.01	0.50 ± 0.02*	0.04	2.90 ± 0.02*	2.41 ± 0.03	1E^−17^	0.032 ± 0.005	0.034 ± 0.007	0.8	13.1 ± 0.2*	10.4 ± 0.2	3E^−13^
Range	0.29 ± 0.01	0.41 ± 0.04*	0.01	0.87 ± 0.03*	0.31 ± 0.04	1E^−14^	0.018 ± 0.05	0.018 ± 0.007	0.9	3.9 ± 0.4*	2.0 ± 0.2	9E^−6^
			Descending Branch									
Parameter	AUC/AUC in 40 mM KCl			Amplitude (g)			Frequency (Hz)					
	V	PB	P	V	PB	P	V	PB	P			
EC50	−7.2 ± 0.3	−7.0 ± 0.3	0.6	−7.6 ± 0.7	−7.3 ± 0.6	0.2	−7.2 ± 0.1	−7.2 ± 0.2	0.9			
Min	0.34 ± 0.01	0.36 ± 0.01	0.4	1.95 ± 0.04*	1.57 ± 0.04	4E^−7^	0.023 ± 0.005	0.021 ± 0.006	0.2			
Max	0.43 ± 0.01	0.52 ± 0.02*	0.02	3.09 ± 0.09*	2.42 ± 0.007	2E^−7^	0.033 ± 0.006	0.035 ± 0.001	0.8			
Range	0.53 ± 0.03	0.54 ± 0.03	0.2	1.1 ± 0.1*	0.85 ± 0.07	0.02	0.010 ± 0.008	0.014 ± 0.001	0.6			
B. Curves parameters for dose response to oxytocin in 1 μM Verapamil												
	Ascending Branch											
Parameter	AUC/AUC in 40 mM KCl			Amplitude (g)			Frequency (Hz)			Duration (s)		
	V	PB	P	V	PB	P	V	PB	P	V	PB	P
EC50	−8.8 ± 0.1	−8.7 ± 0.1	0.6	−10.4 ± 0.2*	−9.5 ± 0.3	0.03	−9.6 ± 0.1	−8.9 ± 0.4	0.3	−8.35 ± 0.5	−8.70 ± 0.1*	2E^−3^
Min	0.05 ± 0.02	0.06 ± 0.02	0.5	1.27 ± 0.04*	0.99 ± 0.02	2E^−8^	0.0148 ± 4E^−4^	0.0210 ± 2E^−3^*	7E^−3^	4.5 ± 0.1*	3.6 ± 0.2	4E^−4^
Max	0.40 ± 0.01	0.50 ± 0.01*	5E^−6^	2.72 ± 0.03*	1.14 ± 0.01	8E^−20^	0.0332 ± 7E^−4^	0.0433 ± 1E^−3^*	3E^−10^	9.29 ± 0.08*	7.2 ± 0.1	2E^−20^
Range	0.35 ± 0.02	0.44 ± 0.03*	7E-7	0.45 ± 0.05*	0.15 ± 0.06	1E^−6^	0.0184 ± 8E^−4^	0.0223 ± 2E^−3^*	7E^−6^	4.8 ± 0.1*	3.6 ± 0.2	2E^−6^
	Descending Branch											
Parameter	AUC/AUC in 40 mM KCl			Amplitude (g)			Frequency (Hz)			Duration (s)		
	V	PB	P	V	PB	P	V	PB	P	V	PB	P
EC50	−8.8 ± 0.1	−8.7 ± 0.1	0.6	−10.4 ± 0.2*	−9.5 ± 0.3	0.03	−9.6 ± 0.1	−8.9 ± 0.4	0.3	−8.35 ± 0.5	−8.70 ± 0.1*	2E^−3^
Min	0.05 ± 0.02	0.06 ± 0.02	0.5	1.27 ± 0.04*	0.99 ± 0.02	2E^−8^	0.0148 ± 4E^−4^	0.0210 ± 2E^−3^*	7E^−3^	4.5 ± 0.1*	3.6 ± 0.2	4E^−4^
Max	0.40 ± 0.01	0.50 ± 0.01*	5E^−6^	2.72 ± 0.03*	1.14 ± 0.01	8E^−20^	0.0332 ± 7E^−4^	0.0433 ± 1E^−3^*	3E^−10^	9.29 ± 0.08*	7.2 ± 0.1	2E^−20^
Range	0.35 ± 0.02	0.44 ± 0.03*	7E-7	0.45 ± 0.05*	0.15 ± 0.06	1E^−6^	0.0184 ± 8E^−4^	0.0223 ± 2E^−3^*	7E^−6^	4.8 ± 0.1*	3.6 ± 0.2	2E^−6^
	Descending Branch											
Paramete				Amplitude (g)			Frequency (g)					
				V	PB	P	V	PB	P			
EC50				−7.6 ± 0.3	−6.6 ± 0.7	0.2	−7.0 ± 0.2	−7.5 ± 0.2	0.5			
Min				1.43 ± 0.03*	1.03 ± 0.04	4E^−10^	0.016 ± 1E^−3^	0.0291 ± 8E^−4^*	4E^−12^			
Max				1.72 ± 0.04*	1.16 ± 0.05	3E^−10^	0.0340 ± 4E^−4^	0.043 ± 1E^−3^*	1E^−10^			
Range				0.29 ± 0.05*	0.13 ± 0.06	0.02	0.018 ± 1E^−3^	0.022 ± 1E^−3^*	3E^−3^			

Note: The parameters of the dose response curves to oxytocin are presented in two groups (ascending and descending branch) to account for the hormetic trend of some of the curves

Frequency curves were also hormetic. In both ascending and descending branches, all parameters except EC50 were higher in PB strips. PB and V frequencies both peaked at 5 nM consistently with the frequency curves of the dose–response to oxytocin in mibefradil. The ascending branches EC50 were similar between groups and consistent with the AUC curves EC50s. This trend is similar to that of the dose response to oxytocin in the absence of inhibitors [1].

In both groups, the event duration of spontaneous contractions was cut in half when 1 μM verapamil rather than 1 μM mibefradil was used (−51 ± 16% in V, −57 ± 14% in PB). Also, the maximal duration was shorter with verapamil than with mibefradil (−29 ± 11% in V, −31 ± 12% in PB). V strips preserved longer durations when treated with verapamil and showed a higher range of response to oxytocin than PB strips throughout the experiment.

### Response to Oxytocin in 10 *µ*M Verapamil

In another set of dose–response to oxytocin experiments, we preconditioned the uterine strips for 20 min with 10 µM verapamil. With this higher dosage of inhibitor, all phasic contractions were completely abolished. The application of increasing oxytocin concentrations resulted in a dose-dependent increase in AUC/AUC in 40 mM KCl, due to an increase in the strips’ basal tone since phasic contractions did not resume.

**Fig. 5:**
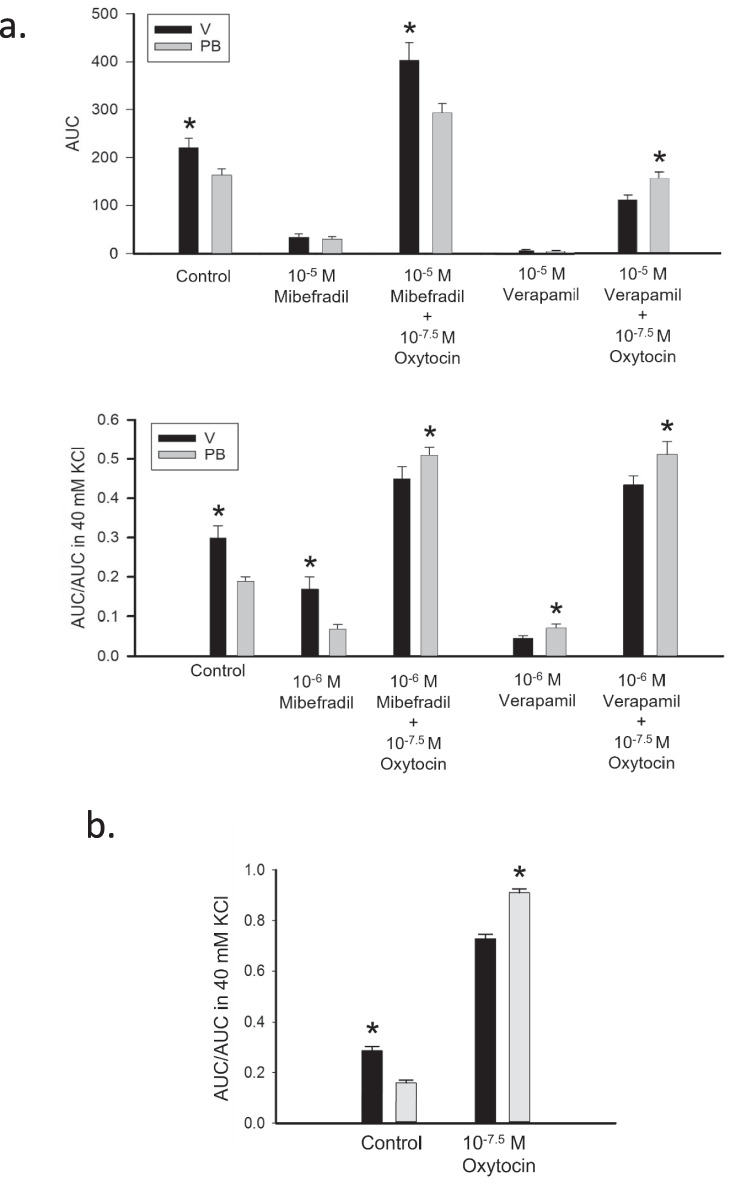
Contractility Histograms. **a.** Top panel: Comparison of raw AUC data of untreated samples (control), in the presence of 10 μM of either mibefradil or verapamil alone and with the addition of 10^–7.5^ M oxytocin. These data originate from inhibition-response experiments. Bottom panel: Comparison of AUC/AUC in 40 mM data of untreated samples (control), in the presence of 1 μM of either mibefradil or verapamil alone and with the addition of 10^–7.5^ M oxytocin. These data originate from the dose–response to oxytocin in the presence of voltage-gated calcium channels blocker experiments. **b.** Reference histogram of AUC/AUC in 40 mM KCl in the presence and absence of oxytocin, according to data previously published by Porta et al. [1]

Under these extreme conditions, the response to oxytocin was still more intense in PB samples than in V samples (see summarizing histograms of Fig. 5 and corresponding data in Table 3). The AUC curves were both sigmoidal. The curve minimum was very similar (0.003 ± 0.001 in PB vs. 0.004 ± 0.002 in V). PB samples showed a higher oxytocin curve maximum and a wider overall response range compared to virgin V samples (Max: 0.106 ± 0.002 vs. 0.077 ± 0.002 in V, *P* = 10^–12^; Range: 0.103 ± 0.003 vs. 0.073 ± 0.003 in V, *P* = 6^–6^). Hence, AUC increased 3.4 folds in PB, 1.8 folds in V. The oxytocin EC50 was identical (−8.36 ± 0.08 for both). The maximal PB strip response to oxytocin in 10 µM verapamil was 88% weaker than the maximal response achieved in the absence of verapamil (0.106 ± 0.002 vs. 0.905 ± 0.02, according to our previously published data [1]) and 78% less robust than the maximal response to oxytocin in the presence of 1 µM verapamil. For V strips, the maximal response to oxytocin in 10 µM verapamil was 90% weaker than the maximal response in the absence of verapamil (0.077. ± 0.002 vs. 0.756 ± 0.001, as per Porta et al. [1]) and 80% less intense than the maximal response with 1 µM verapamil.

**Table 3:** Comparison of response to oxytocin with and without preconditioning with voltage-gated calcium channels inhibitors

	AUC (g * s)					
Condition	V			PB		
	M	SD	SE	M	SD	SE
Control	221*	91	18	163	60	12
10^–5^ Mibefradil	120	71	14	96	42	9
10^–5^ Mibefradil + 10^–7.5^ Oxytocin	403*	179	37	294	95	19
10^–5^ Verapamil	11*	26	5	5	5	3
10^–5^ Verapamil + 10^–7.5^ Oxytocin	109	42	8	134*	50	9
	AUC/AUC in 40 mM KCl					
	V			PB		
	M	SD	SE	M	SD	SE
Control	0.30*	0.16	0.03	0.19	0.08	0.02
10^–6^ Mibefradil	0.17*	0.09	0.01	0.07	0.06	0.01
10^–6^ Mibefradil + 10^–7.5^ Oxytocin	0.45	0.14	0.02	0.53*	0.13	0.02
10^–6^ Verapamil	0.046	0.029	0.005	0.073*	0.041	0.008
10^–6^ Verapamil + 10^–7.5^ Oxytocin	0.43	0.11	0.02	0.51*	0.16	0.03
10^–7.5^ Oxytocin	0.73	0.14	0.03	0.94*	0.13	0.03

Note: Within each set of measures, means with (*) differ significantly (*P* < 0.05). For all conditions except 10^–7.5^ oxytocin, the data were derived from sets of 4 rats (6 strips/rat). The data for the response to 10^–7.5^ oxytocin was derived from sets of 12 rats (6 strips/rat) and reflect previously published results [1]

These data are consistent with the results obtained from the inhibition-response experiment as well as with the results of the dose–response to oxytocin with 1 µM verapamil. Given the absence of phasic contractions, no data on amplitude, frequency, or duration are available for these experiments.

## Discussion

The goal of this study was to investigate the hypotheses that 1) the activity of voltage-gated Ca^2+^ channels (VGCC) is affected by parity and 2) either T-type Ca^2+^ channels (TTCC) or/and L-type Ca^2+^ channels (LTCC) contribute to the differential activation of contraction observed in rat uteri before and after the first pregnancy.

In 2023, we published data supporting the idea that pregnancy induces permanent changes to the contractile properties of the uterus [1]. In that original work, we demonstrated that the spontaneous contractility of uterine samples from female rats that had experienced pregnancy (proven breeders, PB) was weaker than that of uteri from virgin animals. However, the response to oxytocin was stronger in PB uterine samples. These findings suggested that parity affects the uterus’s calcium handling systems, the contractile machinery’s proteins, and/or the number of oxytocin receptors controlling them. In that same study, we started to test the impact of parity on the calcium (Ca^2+^) handling system, by evaluating the responses of V and PB rat uteri to modifications of the extracellular Ca^2+^ availability and sarcoplasmic reticulum (SR) Ca^2+^ release and load mechanisms. We showed that reducing the Krebs solution’s Ca^2+^ content from 2.5 mM to 10 µM both reduced the overall oxytocin response and weakened the parity-related differences in that response. We deduced that at least one of the mechanisms involved in Ca^2+^ entry is likely modified by parity. However, we did not investigate in detail whether VGCCs or store-operated calcium entry (SOCE) channels played a greater part in this mechanism. Here we started addressing the role of VGCCs.

Our first investigative step consisted of inhibition-response experiments to reveal differences in the motility responses to mibefradil and verapamil which could imply variation in the TTCCs and LTCCs availability and functionality.

Spontaneous uterine contractility (expressed as area-under-the-curve, AUC) was more intense in samples from V animals. This correlated mainly to higher amplitudes, as frequencies were slightly higher in PB samples and durations were similar. Contraction amplitude depends directly on intracellular Ca^2+^ level [39]. As expected, the progressive increase in the VGCC inhibitor (mibefradil or verapamil) concentrations reduced amplitudes in both V and PB samples, more strongly in V samples. In the mibefradil experiments, the decline in amplitude started at lower doses in V than in PB samples (although IC50 was not significantly different). This suggests a slightly higher contribution of TTCCs to the V intracellular Ca^2+^ availability. However, we must consider that the selectivity of mibefradil is rather low. The mibefradil IC50 for the TTCC current (I_Ca(T)_) is 0.7 µM, while the mibefradil IC50 for the LTCC current is 2 µM [40]. This means that at the end of the inhibition response curve, with 10 μM mibefradil, TTCCs were likely completely inhibited but a substantial percentage of LTCCs were too. Hence, the possibility that LTCCs might also be more active in V than PB strips cannot be completely ignored, at this point.

At 10 μM mibefradil, contractions still occurred in both groups. Hence, Ca^2+^ entry was sufficient to maintain contraction and preserve at least in part Ca^2+^ stores replenishment. Although the amplitude of these contractions was very similar in V and PB strips, frequency and durations still presented their characteristic differences. At 10 μM verapamil, instead, almost all phasic contractions were eliminated. The rare events in both V and PB samples were uniformly weak and short-lived. It is worth remembering that verapamil’s IC50 is around 0.2 μM for LTCC and about 20 μM for TTCC [32, 41]. Thus, at 10 μM verapamil, all LTCC can be considered fully inhibited, while only some TTCCs are. This is consistent with the greater contribution to calcium entry of LTCCs.

After the last dose of VGCC inhibitor, subsequent stimulation with 50 nM oxytocin caused robust reactivation in both V and PB. However, in the mibefradil experiments, V strips responded more intensely to oxytocin, mainly via longer event durations (force and frequency were similar). This was intriguing because, in our past studies with no VGCC inhibitors, oxytocin was shown to activate more intensely PB uteri, mainly based on a stronger frequency increase.

This new data implicates TTCCs in the mechanism underlying the stronger frequency response of PB tissues to oxytocin. Hence, TTCCs seem to be determinants of the parity-dependent differential response to oxytocin.

In verapamil-treated groups, instead, PB strips responded more robustly. Although duration was still higher in V, 50 nM oxytocin caused a much more intense frequency elevation in PB samples and likely drove the overall effect observed in AUC. This conforms to the observations in the absence of VGCC inhibitors. Thus, suppression of LTCCs currents does not seem to eliminate the differential response of PB to oxytocin, which in turn suggests LTCCs might not be key players in the phenomenon. To confirm these findings, we ran the full dose–response curves to oxytocin in the presence of a given amount of VGCC inhibitor. This provided data we could better compare with results from our previous studies, in which we normalized AUC to the AUC obtained with a 40 mM KCl challenge. While this normalization was necessary to limit the effect of intrinsic strip variations on our measurements, we also showed that the challenge with 40 mM KCl could affect the subsequent behavior of the strips. In addition, in the present VGCC inhibition response experiments, we achieved a 50 nM concentration with a single dose. This differs from our older experiments, where we reached the same concentration through multiple additions over 30–35 min. This procedural change could impact tissue response, as extended oxytocin exposure potentially leads to receptor downregulation.

The AUC/AUC in 40 mM KCl curves for the dose–response to oxytocin in mibefradil experiments showed more spontaneous contractility in V strips and a stronger response (maximum and range) to oxytocin in PB samples, similarly to experiments in the absence of VGCC inhibitors. However, the characteristic difference between V and P AUC curves was visibly reduced. In the dose–response to oxytocin in verapamil experiments, pre-oxytocin contractility was equally low in V and PB samples. Given the predominance of LTCCs and their contribution to the activation of SR Ca^2+^ release, this is not surprising. Again, the maximal activity and range achieved by oxytocin were higher in PB strips. In addition, the overall shape and relative sizes of the curves in the presence of verapamil resembled much more closely the curve obtained in the absence of VGCC. In the presence of mibefradil, the AUC/ACU in 40 mM dose–response to oxytocin curves had a much more pronounced bell shape that could not be fitted satisfactorily with a simple sigmoid. In the absence of VGCC inhibitors, the AUC/ACU in 40 mM dose–response to oxytocin curves were fully sigmoidal at both 2.5 mM and 10 μM Ca^2+^ and well correlated with the sigmoidal shapes of the frequency curves, even if the amplitude curves were hormetic [1]. Since frequency seemed to be the defining factor determining the predominant response to oxytocin in PB, we reasoned that it was likely also the main determinant of the overall shape of the AUC curves. In experiments in which we reduced the SR Ca^2+^ release with ruthenium red (RR), both frequency and AUC curves were bell-shaped. We speculated this could be either an effect of inadequate cytosolic Ca^2+^ availability from the SR at high oxytocin doses, or some non-specific, possible time-dependent action of RR itself. Here, frequency curves were bell-shaped when we preconditioned with either mibefradil or verapamil. Hence, this is not likely a peculiar action of RR but a more general effect connected to pharmacologically altering the function of Ca^2+^ channels. Notably, in the RR experiments, the maximum frequency values elicited by oxytocin were around 0.02 Hz for both V and PB, much lower than either with no pretreatment or with VGCC inhibitors. This underscores the primary importance of SR Ca^2+^ release in uterine contractility. Furthermore, a potential interplay between VGCCs and SR channels might become crucial for sustaining high-frequency contractions at oxytocin levels above 5–10 nM. However, these considerations do not explain why the AUC/AUC in 40 mM KCl curves in verapamil are less hormetic than in RR or mibefradil, despite frequency having strongly hormetic curves. The characteristics of the amplitude and duration curves, therefore, might provide useful insight.

Duration curves present similar sigmoidal trends in both the VGCC inhibitors’ studies, with V samples developing significantly longer contractions than PB samples. Therefore, the differentiating element is likely the variation in amplitude, which directly reflects the cytosolic Ca^2+^ availability. The dose–response to oxytocin always generates bell-shaped amplitude curves, regardless of preconditioning. Yet, the type of preconditioning modulates the curve’s profile, affecting both its width and the oxytocin concentration at which peak amplitude occurs. In mibefradil, oxytocin elicited greater variations in amplitude than in verapamil, especially in V strips. The peak amplitude was achieved at 5 nM mibefradil. Thus, in mibefradil, both the amplitude and the frequency curves peaked at 5 nM oxytocin. Instead, amplitude and frequency peaked at different oxytocin concentrations in verapamil-treated strips. V strips achieved their amplitude peak at 1 nM oxytocin, while PB strips at 50 nM. Yet, frequency peaked at 5 nM oxytocin. This likely accounts for the more pronounced bell shape of the overall oxytocin AUC/AUC in 40 mM KCl in the presence of mibefradil.

In general, preconditioning with verapamil greatly limited the ability of oxytocin to modify amplitude. The bell-shaped amplitude curves in verapamil appear flattened in both V and PB, although more so in the latter. In our earlier experiments with no VGCC inhibitors, PB strips showed a stronger increase in amplitude in response to oxytocin than V strips, although the latter had significantly higher overall amplitude. When extracellular Ca^2+^ was reduced to 10 µM, PB strips’ amplitude increased less than V strips. The same pattern occurs upon inhibition with mibefradil and verapamil. These observations support our earlier conclusion that more TTCCs might be available for activation in V samples. Mibefradil’s limited selectivity for TTCCs hinders definitive conclusions about LTCCs predominance in V or PB uteri.

Finally, although V might have a vaster contingent of TTCCs, their relative distribution in V and PB myometrial cells warrants further consideration. Preconditioning with mibefradil caused the frequency of oxytocin response curves to be similar in V and PB. This is a crucial detail, based on the presumed role of frequency in the differential response of V and PB to oxytocin. Besides the overall shape of the frequency curve, there was no significant difference in maximal response and range between groups. In addition, these maximal frequencies were like those recorded in V strips without VGCC inhibitors and with verapamil. Hence, it appears that, regardless of their numbers in V vs. PB, TTCCs are important determinants of the stronger frequency response in PB strips.

T-type calcium channels (TTCCs) play a crucial role in the rhythmic activity of pacemaker cells in both the heart and certain smooth muscles. In the heart’s sinoatrial node, TTCCs contribute to the spontaneous depolarization that initiates each heartbeat. Their low activation threshold allows them to contribute to the "pacemaker potential," driving the sinoatrial node cells toward the threshold for action potential firing [42]. In smooth muscles, such as those found in blood vessels, TTCCs can contribute to rhythmic contractions that regulate vascular tone (Cribb, 2004). Additionally, TTCCs found in some intestinal smooth muscles may participate in the generation of slow waves, contributing to gut motility [43, 44].

Unlike the heart, with its well-defined sinoatrial node as the central pacemaker, the uterus appears to lack a single, centralized pacemaker region [45] [46]. Instead, research suggests the presence of specialized cells scattered throughout the myometrium that exhibit pacemaker-like qualities [47]. These cells can spontaneously generate electrical activity, potentially initiating uterine contractions. However, the exact location and distribution of these putative pacemaker cells remain a subject of ongoing investigation. Some studies suggest that potential pacemaker regions may be concentrated around the placental bed and near vascular networks [45].

Based on these considerations and the data presented here, we speculate that, following pregnancy, pacemaker cells of the uterus express a higher concentration of TTCCs that contributes to the stronger increase in frequency in response to oxytocin observed in the PB uterus. This explanation harmonizes the findings on the larger impact of VGCC in V uteri with the evidence on the important role of TTCCs in the frequency-driven, more robust response of PB myometrial samples to oxytocin.

In conclusion, this study presents evidence supporting the role of voltage-gated calcium channels (VGCCs) in mediating the differential oxytocin response observed between nulliparous and parous uteri. Our findings indicate that nulliparous uteri possess a greater abundance of VGCCs, notably T-type calcium channels. Conversely, parous uteri demonstrate a stronger frequency response to oxytocin, which we suggest is linked to a more significant role for T-type calcium channels in their myometrial pacemaker cells. These results highlight the potential influence of both VGCC quantity and their precise function within uterine cells as key factors determining the contractile characteristics of nulliparous and parous uterine tissues.

To further dissect the mechanisms underlying the differential response to oxytocin in V and PB samples, our subsequent research steps will involve both functional and molecular investigations. Motility studies will directly examine the role of ORAI and TRP channels by utilizing specific pharmacological inhibitors of these channels. These experiments will reveal whether selective blockade of ORAI or TRP channels differentially affects oxytocin-induced contractions in V and PB tissues. Alongside these studies, we will conduct molecular biology analyses to quantify the protein expression levels of key VGCCs and SOCE channels in both V and PB myometrium. This approach will provide crucial insights into potential variations in the abundance of these channels, offering a molecular basis for the observed functional differences in oxytocin response. Further investigations will be crucial to fully elucidate how the interplay between VGCC expression, pacemaker cell activity, and the uterine physiological state shape labor patterns. The idea that T-type calcium channels (TTCCs) play a heightened role in the parous uterus, leading to a stronger frequency response to oxytocin, provides a potential link to the increased risk of premature labor associated with prior pregnancies. Could an overly sensitive oxytocin response, driven by TTCCs, initiate contractions prematurely in subsequent pregnancies? Might this explain the higher susceptibility to preterm labor in women with previous births [48]? While these questions require further exploration, our results emphasized that understanding how pregnancy reshapes the uterine response to calcium could be key to preventing premature birth.

The clinical implications of this research are significant. By identifying the specific calcium channels and signaling pathways involved in preterm labor, the development of targeted therapies that modulate uterine contractility may be possible, potentially preventing early contractions without affecting the fetus or normal labor. Understanding the molecular changes in the uterus after childbirth could enable personalized risk assessments for premature birth, allowing healthcare providers to tailor care plans for women with prior pregnancies.

In conclusion, this study represents not only a scientific advancement but also a step towards improving maternal and neonatal health outcomes.

## Data Availability

Not applicable.

## Declarations

### Animal Research

IACUC Protocol #2758 with IACUC committee approval included.

## References

[1] Porta M, Boening A, Tiemann J, Zack A, Patel A, Sondgeroth K. The contractile response to oxytocin in non-pregnant rat uteri is modified after the first pregnancy. Reprod Sci. 2023;30(7):2152–65. 10.1007/s43032-023-01163-6. 36696040 10.1007/s43032-023-01163-6 PMC10310576

[2] Wray S, Arrowsmith S. Uterine excitability and ion channels and their changes with gestation and hormonal environment. Annu Rev Physiol. 2021;83(1):331–57. 10.1146/annurev-physiol-032420-035509. 33158376 10.1146/annurev-physiol-032420-035509

[3] Nowycky MC, Fox AP, Tsien RW. Three types of neuronal calcium channel with different calcium agonist sensitivity. Nature. 1985;340:233–6. 10.1038/316440a0. 10.1038/316440a0 2410796

[4] Fox AP, Nowycky MC, Tsien RW. Kinetic and pharmacological properties distinguishing three types of calcium currents in chick sensory neurones. J Physiol. 1987;394:149–72. 10.1113/jphysiol.1987.sp016864. 2451016 10.1113/jphysiol.1987.sp016864 PMC1191955

[5] Huguenard JR. Low-threshold calcium currents in central nervous system neurons. Annu Rev Physiol. 1996;58(1):329–48. 10.1146/annurev.ph.58.030196.001553. 8815798 10.1146/annurev.ph.58.030196.001553

[6] Hagiwara N, Irisawa H, Kameyama M. Contribution of two types of calcium currents to the pacemaker potentials of rabbit sino-atrial node cells. J Physiol. 1988;395(1):233–53. 10.1113/jphysiol.1988.sp016916. 2457676 10.1113/jphysiol.1988.sp016916 PMC1191991

[7] Rossier MF. Function and differential expression of T-type calcium channels in various pathophysiological states. Recent Res Dev Biochem. 2003;4:13–29.

[8] Rossier MF. T-Type calcium channel: a privileged gate for calcium entry and control of adrenal steroidogenesis. Front Endocrinol. 2016;7:43. 10.3389/fendo.2016.00043. 10.3389/fendo.2016.00043 PMC4873500 27242667

[9] Perez-Reyes E. Molecular physiology of low-voltage-activated t-type calcium channels. Physiol Rev. 2003;83(1):117–61. 10.1152/physrev.00018.2002. 12506128 10.1152/physrev.00018.2002

[10] Young RC, Smith LH, McLaren MD. T-type and L-type calcium currents in freshly dispersed human uterine smooth muscle cells. Am J Obstet Gynecol. 1993;169(4):785–92. 10.1016/0002-9378(93)90006-5. 8238133 10.1016/0002-9378(93)90006-5

[11] Blanks AM, Zhao ZH, Shmygol A, Bru-Mercier G, Astle S, Thornton S. Characterization of the molecular and electrophysiological properties of the T-type calcium channel in human myometrium. J Physiol. 2007;581(3):915–26. 10.1113/jphysiol.2007.132126. 17446221 10.1113/jphysiol.2007.132126 PMC1976399

[12] Ohkubo T, Inoue Y, Kawarabayashi T, Kitamura K. Identification and electrophysiological characteristics of isoforms of T-type calcium channel Ca(v)3.2 expressed in pregnant human uterus. Cell Physiol Biochem. 2005;16(4–6):245–54. 10.1159/000089850. 16301824 10.1159/000089850

[13] Ohkubo T, Kawarabayashi T, Inoue Y, Kitamura K. Differential expression of L- and T-type calcium channels between longitudinal and circular muscles of the rat myometrium during pregnancy. Gynecol Obstet Invest. 2005;59(2):80–5. 10.1159/000082333. 15564792 10.1159/000082333

[14] Lee SE, Ahn DS, Lee YH. Role of T-type Ca channels in the spontaneous phasic contraction of pregnant rat uterine smooth muscle. Korean J Physiol Pharmacol. 2009;13(3):241–9. 10.4196/kjpp.2009.13.3.241. 19885043 10.4196/kjpp.2009.13.3.241 PMC2766731

[15] Bermejo PE, Anciones B. A review of the use of zonisamide in Parkinson’s disease. Ther Adv Neurol Disord. 2009;2(5):313–7. 10.1177/1756285609338501. 21180621 10.1177/1756285609338501 PMC3002602

[16] Dziegielewska B, Gray LS, Dziegielewski J. T-type calcium channels blockers as new tools in cancer therapies. Pflugers Arch. 2014;466(4):801–10. 10.1007/s00424-014-1444-z. 24449277 10.1007/s00424-014-1444-z

[17] Zhang L, Wang L, Jiang J, Zheng D, Liu S, Liu C. Lipopolysaccharides upregulate calcium concentration in mouse uterine smooth muscle cells through the T-type calcium channels. Int J Mol Med. 2015;35(3):784–90. 10.3892/ijmm.2014.2054. 25573237 10.3892/ijmm.2014.2054

[18] Kopecky BJ, Liang R, Bao J. T-type calcium channel blockers as neuroprotective agents. Pflugers Arch. 2014;466(4):757–65. 10.1007/s00424-014-1454-x. 24563219 10.1007/s00424-014-1454-x PMC4005039

[19] He L, Yu Z, Geng Z, Huang Z, Zhang C, Dong Y, Gao Y, Wang Y, Chen Q, Sun L, Ma X, Huang B, Wang X, Zhao Y. Structure, gating, and pharmacology of human CaV3.3 channel. Nat Commun. 2022;13(1):2084. 10.1038/s41467-022-29728-0. 35440630 10.1038/s41467-022-29728-0 PMC9019099

[20] Morales D, Hermosilla T, Varela D. Calcium-dependent inactivation controls cardiac L-type Ca^2+^ currents under β-adrenergic stimulation. J Gen Physiol. 2019;151(6):786–97. 10.1085/jgp.201812236. 30814137 10.1085/jgp.201812236 PMC6571991

[21] Grandi E, Morotti S, Ginsburg KS, Severi S, Bers DM. Interplay of voltage and Ca-dependent inactivation of L-type Ca current. Prog Biophys Mol Biol. 2010;103(1):44–50. 10.1016/j.pbiomolbio.2010.02.001. 20184915 10.1016/j.pbiomolbio.2010.02.001 PMC2907421

[22] Catterall WA. Voltage-gated calcium channels. Cold Spring Harb Perspect Biol. 2011;3(8):a003947. 10.1101/cshperspect.a003947. 21746798 10.1101/cshperspect.a003947 PMC3140680

[23] Lipscombe D, Helton TD, Xu W. L-type calcium channels: the low down. J Neurophysiol. 2004;92(5):2633–41. 10.1152/jn.00486.2004. 15486420 10.1152/jn.00486.2004

[24] Feng T, Kalyaanamoorthy S, Barakat K (2018) L-Type calcium channels: structure and functions [Internet]. Ion Channels in Health and Sickness. InTech. Available from: 10.5772/intechopen.77305

[25] Wegener JW, Schulla V, Koller A, Klugbauer N, Feil R, Hofmann F. Control of intestinal motility by the Ca(v)1.2 L-type calcium channel in mice. Faseb J. 2006;20(8):1260–2. 10.1096/fj.05-5292fje. 16636102 10.1096/fj.05-5292fje

[26] Ghosh D, Syed AU, Prada MP, Nystoriak MA, Santana LF, Nieves-Cintrón M, Navedo MF. Calcium channels in vascular smooth muscle. Adv Pharmacol. 2017;78:49–87. 10.1016/bs.apha.2016.08.002. 28212803 10.1016/bs.apha.2016.08.002 PMC5439506

[27] Reynolds NA, Wagstaff AJ, Keam SJ. Trandolapril/verapamil sustained release: a review of its use in the treatment of essential hypertension. Drugs. 2005;65(13):1893–914. 10.2165/00003495-200565130-00011. 16114984 10.2165/00003495-200565130-00011

[28] Kato M, Dote K, Sasaki S, Takemoto H, Habara S, Hasegawa D. Intracoronary verapamil rapidly terminates reperfusion tachyarrhythmias in acute myocardial infarction. Chest. 2004;126(3):702–70. 10.1378/chest.126.3.702. 15364745 10.1378/chest.126.3.702

[29] Kirchhof P, Benussi S, Kotecha D, Ahlsson A, Atar D, Casadei B, Castella M, Diener HC, Heidbuchel H, Hendriks J, Hindricks G, Manolis AS, Oldgren J, Popescu BA, Schotten U, Van Putte B, Vardas P, ESC Scientific Document Group. 2016 ESC Guidelines for the management of atrial fibrillation developed in collaboration with EACTS. Eur Heart J. 2016;37(38):2893–962. 10.1093/eurheartj/ehw210. 27567408 10.1093/eurheartj/ehw210

[30] Nogami A. Purkinje-related arrhythmias part I: monomorphic ventricular tachycardias. Pacing Clin Electrophysiol. 2011;34(5):624–50. 10.1111/j.1540-8159.2011.03044.x. 21410719 10.1111/j.1540-8159.2011.03044.x

[31] Hockerman GH, Johnson BD, Abbott MR, Scheuer T, Catterall WA. Molecular determinants of high affinity phenylalkylamine block of L-type calcium channels in transmembrane segment IIIS6 and the pore region of the alpha1 subunit. J Biol Chem. 1997;272(30):18759–65. 10.1074/jbc.272.30.18759. 9228049 10.1074/jbc.272.30.18759

[32] Bergson P, Lipkind G, Lee SP, Duban ME, Hanck DA. Verapamil block of T-type calcium channels. Mol Pharmacol. 2011;79(3):411–9. 10.1124/mol.110.069492. 21149638 10.1124/mol.110.069492 PMC3061365

[33] Zhang S, Zhou Z, Gong Q, Makielski JC, January CT. Mechanism of block and identification of the verapamil binding domain to HERG potassium channels. Circ Res. 1999;84(9):989–98. 10.1161/01.res.84.9.989. 10325236 10.1161/01.res.84.9.989

[34] Xynogalos P, Rahm AK, Fried S, Chasan S, Scherer D, Seyler C, Katus HA, Frey N, Zitron E. Verapamil inhibits Kir2.3 channels by binding to the pore and interfering with PIP2 binding. Naunyn Schmiedebergs Arch Pharmacol. 2023;396(4):659–67. 10.1007/s00210-022-02342-z. 36445385 10.1007/s00210-022-02342-z PMC10042922

[35] Herrera-Pérez S, Rueda-Ruzafa L, Campos-Ríos A, Fernández-Fernández D, Lamas JA. Antiarrhythmic calcium channel blocker verapamil inhibits trek currents in sympathetic neurons. Front Pharmacol. 2022;13:997188. 10.3389/fphar.2022.997188. 36188584 10.3389/fphar.2022.997188 PMC9522527

[36] Marcondes FK, Bianchi FJ, Tanno AP. Determination of the estrous cycle phases of rats: some helpful considerations. Braz J Biol. 2002;62:609–14. 10.1590/s1519-69842002000400008. 12659010 10.1590/s1519-69842002000400008

[37] Miyashita-Ishiwata M, El Sabeh M, Reschke LD, Afrin S, Borahay MA. Differential response to hypoxia in leiomyoma and myometrial cells. Life Sci. 2022;290:120238. 10.1016/j.lfs.2021.120238. 34942165 10.1016/j.lfs.2021.120238 PMC8757389

[38] Alotaibi M, Arrowsmith S, Wray S. Hypoxia-induced force increase (HIFI) is a novel mechanism underlying the strengthening of labor contractions, produced by hypoxic stresses. Proc Natl Acad Sci U S A. 2015;112(31):9763–8. 10.1073/pnas.1503497112. 26195731 10.1073/pnas.1503497112 PMC4534208

[39] Arrowsmith S, Keov P, Muttenthaler M, Gruber CW. Contractility measurements of human uterine smooth muscle to aid drug development. J Vis Exp. 2018;131:e56639. 10.3791/56639. 10.3791/56639 PMC5841565 29443077

[40] Alcock J, Warren AY, Goodson YJ, Hill SJ, Khan RN, Lymn JS. Inhibition of tissue transglutaminase 2 attenuates contractility of pregnant human myometrium. Biol Reprod. 2011;84(4):646–53. 10.1095/biolreprod.110.085506. 21123816 10.1095/biolreprod.110.085506

[41] Duan JJ, Ma JH, Zhang PH, Wang XP, Zou AR, Tu DN. Verapamil blocks HERG channel by the helix residue Y652 and F656 in the S6 transmembrane domain. Acta Pharmacol Sin. 2007;28(7):959–67. 10.1111/j.1745-7254.2007.00562.x. 17588331 10.1111/j.1745-7254.2007.00562.x

[42] Lakatta EG, Maltsev VA, Vinogradova TM. A coupled SYSTEM of intracellular Ca2+ clocks and surface membrane voltage clocks controls the timekeeping mechanism of the heart’s pacemaker. Circ Res. 2010;106(4):659–73. 10.1161/CIRCRESAHA.109.206078. 20203315 10.1161/CIRCRESAHA.109.206078 PMC2837285

[43] Zheng H, Park KS, Koh SD, Sanders KM. Expression and function of a T-type Ca2+ conductance in interstitial cells of Cajal of the murine small intestine. Am J Physiol Cell Physiol. 2014;306(7):C705–13. 10.1152/ajpcell.00390. 24477235 10.1152/ajpcell.00390.2013 PMC3962600

[44] Ward SM, Dixon RE, de Faoite A, Sanders KM. Voltage-dependent calcium entry underlies propagation of slow waves in canine gastric antrum. J Physiol. 2004;561(3):793–810. 10.1113/jphysiol.2004.076067. 15498805 10.1113/jphysiol.2004.076067 PMC1665383

[45] Lutton EJ, Lammers WJEP, James S, van den Berg HA, Blanks AM. Identification of uterine pacemaker regions at the myometrial-placental interface in the rat. J Physiol. 2018;596(14):2841–52. 10.1113/JP275688. 29704394 10.1113/JP275688 PMC6046083

[46] Wray S, Burdyga T, Noble D, Noble K, Borysova L, Arrowsmith S. Progress in understanding electro-mechanical signalling in the myometrium. Acta Physiol (Oxf). 2015;213(2):417–31. 10.1111/apha.12431. 25439280 10.1111/apha.12431

[47] Young RC. The uterine pacemaker of labor. Best Pract Res Clin Obstet Gynaecol. 2018;52:68–87. 10.1016/j.bpobgyn.2018.04.002. 29866432 10.1016/j.bpobgyn.2018.04.002

[48] Ytterberg K, Jacobsson B, Flatley C, Juodakis J, Nilsson S, Solé-Navais P. Exploring the association of parity and its interaction with history of preterm delivery on gestational duration. Ann Epidemiol. 2023;87:60–8. 10.1016/j.annepidem.2023.09.004. 10.1016/j.annepidem.2023.09.004 37714417

